# The Use of Structural Allograft in Primary and Revision Knee Arthroplasty with Bone Loss

**DOI:** 10.4061/2011/578952

**Published:** 2011-09-26

**Authors:** Raul A. Kuchinad, Shawn Garbedian, Benedict A. Rogers, David Backstein, Oleg Safir, Allan E. Gross

**Affiliations:** Department of Orthopaedic Surgery, Mount Sinai Hospital, Toronto, ON, Canada M5G 1X5

## Abstract

Bone loss around the knee in the setting of total knee arthroplasty remains a difficult and challenging problem for orthopaedic surgeons. There are a number of options for dealing with smaller and contained bone loss; however, massive segmental bone loss has fewer options. Small, contained defects can be treated with cement, morselized autograft/allograft or metal augments. Segmental bone loss cannot be dealt with through simple addition of cement, morselized autograft/allograft, or metal augments. For younger or higher demand patients, the use of allograft is a good option as it provides a durable construct with high rates of union while restoring bone stock for future revisions. Older patients, or those who are low demand, may be better candidates for a tumour prosthesis, which provides immediate ability to weight bear and mobilize.

## 1. Introduction

Dealing with bone loss when performing primary or revision total knee arthroplasty is a challenge for the arthroplasty surgeon. Previous infections, tumour, and trauma can all result in bone loss that makes a standard primary total knee arthroplasty impossible without restoration of bone stock. More commonly, bone loss in revision knee arthroplasty is a frequent problem and may occur for any of the aforementioned reasons, osteolysis, periprosthetic fracture, or iatrogenically when components are being removed from host bone. 

Patients with posttraumatic osteoarthritis or deformity requiring knee arthroplasty often have bone loss in the tibia, femur, or both. In this situation, the surgeon must determine the extent of bone loss and whether it may be dealt with by simple autogenous bone grafting, cement, metal augments, porous metal supplementation, or allograft of various sizes. Large uncontained defects of the knee may be treated with use of a large or massive allograft in conjunction with the total knee.

## 2. Classification

There is no universally accepted classification that is currently used for describing bone loss in knee arthroplasty. Engh developed the Anderson Orthopaedic Research Institute (AORI) classification system that helps to guide treatment for both femoral and tibial sides in revision knee arthroplasty (see Table 1) [1]. 

Mount Sinai Hospital in Toronto, Canada, has developed a classification system, which simply divides the defects into contained or uncontained categories to be used in the arthroplasty setting (see Table 2) [2–8].

Both classification systems attempt to characterize the defects present and assist the surgeon in developing a treatment algorithm for dealing with bone loss, although the AORI classification is more explicit in detailing various treatment options.

## 3. Allograft Characteristics

Allograft harvesting should be done according to the criteria of the American Association of Tissue Banks, in sterile conditions and in our institution followed by irradiating the tissue at 25,000 Gy and storage at −70°C [9]. Although some believe that donor allograft does not have to be matched to the recipient's anatomy, others argue that modifying the allograft weakens it. If the allograft is size matched, application of the graft becomes easier to use in the patient and maintains its inherent strength. Also, allografts that are oversized may make the soft-tissue closure difficult or impossible to perform which is a serious intraoperative complication. To ensure this does not happen, we recommend taking preoperative calibrated radiographs of the allograft and comparing this with the patient's radiographs [2].

## 4. Indications

**Figure 1:**
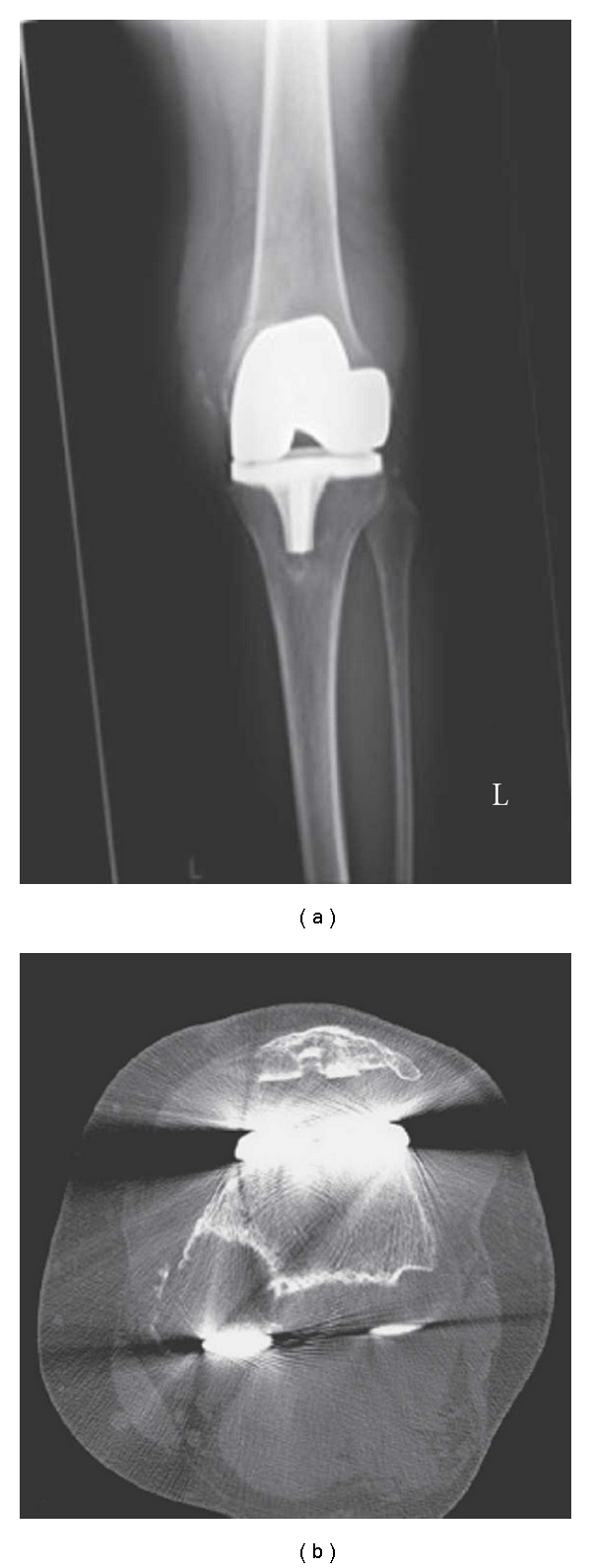
AP radiograph showing a knee with severe polyethylene wear and evidence of major bone loss (a). A CT scan showing massive bone loss of the medial and lateral femoral condyles due to osteolysis (b). (reprinted from Backstein et al. [2]).

**Figure 2:**
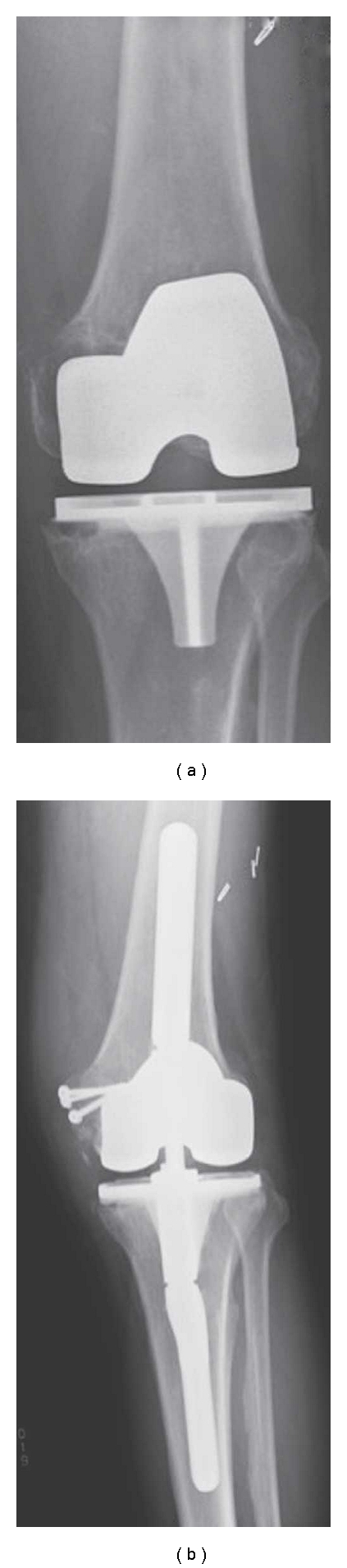
A radiograph shows uncontained bone loss in the medial femoral condyle secondary to osteolysis (a). A radiograph showing revision TKA with reconstruction of the medial femoral condyle using structural allograft fixed with screws (b).

The primary indications for using structural allografts in the setting of arthroplasty are (a) large uncontained defects that are outside the range of metal augments or thicker polyethylene inserts (see Figures 1 and 2), (b) patients that are active and require bone-stock restoration for potential future operations, and (c) patients who are physically well enough to tolerate both the surgical procedure and rehabilitation required for successful outcomes. A relative contraindication is a patient actively smoking, and cessation programs must be implemented prior to surgery. Lastly, presence of active infection is an absolute contraindication for allograft in the arthroplasty patient. 

## 5. Preoperative Preparation and Planning

In the setting of previous infection or posttraumatic defects, active infection must be ruled out. C-reactive Protein, erythrocyte sedimentation rate, and possible knee aspirate should be performed prior to planning any knee arthroplasty procedure especially with use of allografts. Once infection is ruled out, careful planning should include 4 foot standing radiographs of both limbs, standard AP, lateral, and skyline views and, if required, a CT scan. CT scanning can help with determination of whether the defect is contained or uncontained and overall dimensions. As always, these investigations must be combined with a thorough physical exam of the patient, which includes limb alignment, ligamentous stability, and a neurovascular exam.

Preoperative planning incorporates all aspects of the physical exam and investigations but also entails determining surgical approach, dealing with difficult exposure, allograft availability, and arthroplasty component selection. When massive allografts are used, a stemmed implant is required to obtain adequate stability of the component between the host-allograft bone junction. Furthermore, if there is significant ligamentous instability, there should be implants available with higher degrees of constraint.

## 6. Operative Techniques

Old operative reports detailing prior surgical approaches should be obtained to help the surgeon decide on the optimal exposure. Ideally, use of a midline incision and a parapatellar arthrotomy (medial or lateral) should be reused in the revision surgery to minimize the remaining blood supply to the skin and patella.

During the exposure, presence of scar tissue, quadriceps, and patellar tendon contracture and deformity must be adequately dealt with to assist in performing the procedure. Tibial tubercle osteotomy, quadriceps snip, lateral parapatellar arthrotomy, and in situ bony cuts and removal of accessible implants are a few of the adjuncts that can help the surgeon with exposure. It is critical to avoid excessive disruption of the soft-tissue envelope, as wound problems can be a frequent complication of these complex reconstructions [10].

During exposure and debridement, a frozen section should be sent to the pathologist to rule out infection. We typically use a count of less than 5 neutrophils per high power field as a negative result [11]. If infection is suspected or confirmed, the planned surgical procedure is abandoned and a dynamic or static spacer with antibiotic impregnated cement is used until the infection is cleared.

Debridement of nonviable bone and necrotic tissue should also be done during the exposure. The level of debridement should be done to expose healthy bleeding tissue. Implants are removed with microoscillating saws, gigli saws, flexible osteotomes, or through osteotomies. This part of the procedure should be done with care as creating further bone loss increases the complexity of the reconstruction. Furthermore, the quality of the host bone is often osteoporotic and fragile from prior infection, osteolysis, or disuse.

Once exposure is completed, the area of bone loss should be evaluated and classified to determine the type of allograft required for treatment. Ideally, the intraoperative findings should not be unexpected and simply confirm the pathology that was seen in preoperative imaging.

## 7. Segmental Allografts

Small contained defects less than 10 mm can be treated with morselized autograft, allograft, or cement alone. Uncontained defects that are less than 10–20 mm in size can be treated with metal augments alone; however, larger defects can be dealt with structural allograft or tumour implants [12]. Bone loss of the proximal tibia that involves the entire surface can be treated with metal augments and a thicker polyethylene insert, but the upper limit for this is 45 mm. An alternative option is structural allograft or tumour prosthesis.

If a structural allograft is going to be used, having two surgical teams present is ideal. This decreases the anaesthetic time the patient must endure and is the most efficient use of operating room time. One surgical team should have a sterile back table available to prepare the allograft, while the other team simultaneously does the exposure and bony preparation of the patient. 

The major principles of the revision are to determine the level of the joint line that should be measured from the distal femur or proximal fibula. Typically intact host bone is easier to judge where the true joint line should exist. From the medial epicondyle, the joint line is 25–30 mm distal, and, from the tip of the fibula, it is 10–15 mm proximal. Occasionally intraoperative radiographs of the affected and normal knee may be utilized to find the anatomic joint line. Ligamentous structures must also be evaluated to determine whether or not further constraint will be required in the implants. The surgeon must be careful to preserve these attachments during the exposure, debridement, and implant removal.

The goal of the reconstruction should also include balancing the flexion and extension gaps to have a good functional outcome for the patient. Appropriate bone resection and trial implantation position are critical in obtaining this intraoperatively.

The tibial and femoral canals are reamed to have good press fit for trial stems. If needed, offset stems can be used to better align the femoral and tibial trays. Once the trial implants are appropriately positioned, the amount of bone loss should be reevaluated. Irregularly shaped areas of segmental bone loss that is too large for metal augments can be treated with structural allograft. These areas should be made into more geometric defects with the use of precise cutting guides or freehand with an oscillating or reciprocating saw. Once the defects are reshaped, preferably into a square or rectangular shape, they are measured for the height and width. On the back table, the allograft is cut into almost identical size, but slightly larger. We prefer to use bone from the donor that is from the same anatomic region. Osteoporotic allograft bone should be avoided, as this does not have the structural integrity required for support of the implant. If the geometry allows it, a press fit into the defect can be achieved. Certain cases of bone loss caused by infection or osteolysis may result in mixed contained-uncontained defects that can be treated with the press-fit technique. The locations of these areas of bone loss are frequently located at the implant-host interface near the joint line or between medial and lateral columns of the distal femur. In our experience, the addition of supplementary plate fixation does not enhance the allograft stability and may result in a stress riser,due to the additional stiffness, if a plate was placed near the allograft-host bone interface. [Editorial: Meaning extra screw holes through the plate weakens the allograft].

 There are certain circumstances when the press-fit of the allograft into a defect is not sufficient and fixation is required. The technique we prefer to employ is to place the allograft into the desired position and place provisional K-wires. We then continue our reaming and preparation of the trial implants with the allograft in situ. Placement of definitive fixation in the form of cancellous screws with washers should be done with the trial stems in place. This must be done to avoid screws blocking the path of the final stemmed implant. The use of a stemmed implant is critical as it shields the allograft from excessive force. Once the allograft is secured we recheck all bony cuts prior to implanting the definitive prosthesis.

## 8. Allograft-Prosthetic Composites

Massive segmental bone loss of either the femur or tibia cannot be treated with cement, augments, or segmental allograft bone alone and require an allograft-prosthetic component (APC) or tumour prosthesis. These defects are uncontained and are frequently circumferential and involve >25 mm of the femur or >45 mm of the tibia.

After the failed implant is removed and debridement completed, the defects are once again evaluated. If it is decided that a femoral allograft-prosthetic composite is required, the collateral ligaments must be maintained as previously mentioned. Ideally the epicondyle attachments are removed with some host bone present for later reattachment to the allograft. Once this is done, an oblique cut is made in the host bone where the prosthetic-composite interface is to be. Alternatively, a step cut may be employed with the longer limb on the host bone side ideally. This may be slightly more challenging to perform and accurately match the host graft interface. Regardless, either an oblique cut or step cut provides good rotational control of the allograft. If this is not feasible, the allograft may be intussuscepted into the host diaphysis if the host canal is patulous. This telescoping of the two interfaces imparts some stability and increases the contact area between the host allograft that may improve the ability of the allograft to incorporate [2].

## 9. Tibial Allograft-Prosthetic Composite

The allograft-prosthetic composite of the tibia is fashioned to size based on careful measurements of the host tibia after a thorough debridement is performed. As always, making the allograft larger and longer than may actually be required is good practice as it is always easier to trim the graft “down to size” if needed. This saves time and avoids unnecessary waste of allograft. As in any stemmed implant, the host canal is reamed to secure a press-fit stem that should bypass the allograft-host junction by two cortical diameters or by approximately 5 cm. The proximal extent of the allograft should restore the normal biomechanics of the knee and that ultimately means the joint line of the implant should be 10–15 mm proximal to the tip of the fibular head. Again, use of the step or oblique cut is utilized to optimize the stability of the implant. Rotational position is a challenge to determine; however, use of anatomical landmarks such as the tibial tubercle, patellar tendon, and patellar tracking all assist the surgeon in placing the allograft in the correct rotation. This rotational and joint-line position should be judged with the trial implants in place. The knee should be taken through a range of motion to examine the patellar height and tracking. Minor adjustments can be made easily at this stage to improve the knee biomechanics. When the surgeon is satisfied with the rotation and height, the position should be marked with cautery and a marking pen. This assists in final implantation of the APC into the proper overall orientation.

## 10. Femoral Allograft-Prosthetic Composite

**Figure 3:**
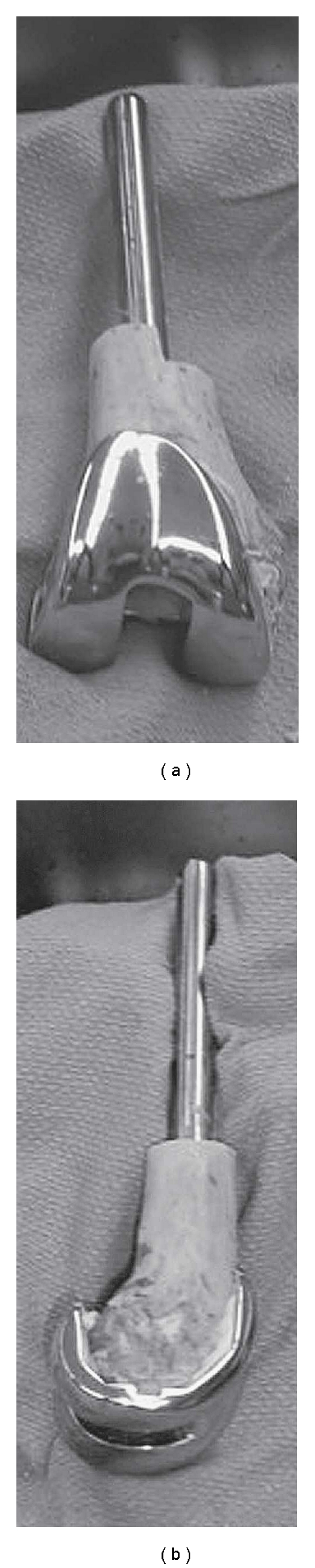
Intraoperative pictures of allograft-prosthesis composite (APC), AP view (a) and lateral view (b).

The epicondylar attachments of the collaterals, which were preserved during exposure, are critical in the securing of the femoral APC. As in the tibia, the femoral canals are reamed to securely fit a stemmed implant with proximal fixation into the host bone of two cortical diameters or a minimum of 5 cm. On the back table, the femoral APC is prepared with the revision cutting guides to make the appropriate bone resections (see Figure 3). The epicondylar attachments of the collaterals are secured to the allograft through transosseous drill-hole tunnels where the collateral ligaments would be in a native distal femur. Sutures are passed through these tunnels and left long to attach the host collaterals once the APC is implanted.

The trial femoral components with their securely fitted stems are implanted into the host diaphysis. The flexion and extension gaps are checked and adjusted as needed. If the extension gap is tight, distal femoral resection of the allograft is performed, and, if the flexion gap is tight, the components are translated anteriorly or downsized. If both flexion and extension gaps are tight, we recommend adjusting cuts on the femoral side and downsizing rather than taking any more of the native proximal tibia. This will also ensure that overstuffing of the knee does not occur and makes wound closure less difficult.

**Figure 4:**
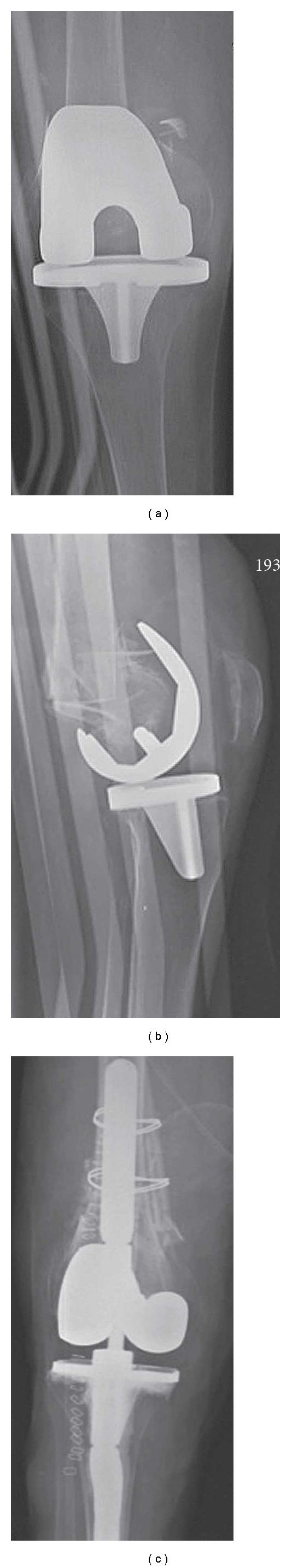
Radiograph showing a supracondylar periprosthetic fracture with major bone loss ((a) and (b)). An AP radiograph showing a revision with femoral allograft-implant composite (c).

When it is time to implant the stems of either the femoral or tibial side, a critical principle is to avoid cementing of the stems to the host bone. Conversely the allograft side of the stem and implant-allograft interface *must be cemented *to provide stability to construct. Meticulous cement technique needs to be utilized to ensure the allograft-implant interface has the requisite stability to allow early motion and rehabilitation (see Figure 4). Thus, a copiously irrigated allograft, which is carefully dried, is requisite prior to cementing. Use of low-dose antibiotic containing cement is acceptable; however, we do not add additional antibiotic to the cement as it weakens it and may potentially result in a poor cement mantle. The cement is allowed to harden the APC, and, once this is done, it is implanted into the host canal through a press fit. Rotational position should be aligned to the previous cautery or marker line as it is impacted. No cement should be present between the allograft-host bone junction as it would potentially interfere with graft incorporation. We emphasize avoidance of cementing stems to the host bone as it can make future revisions extremely difficult.

Once components are implanted, the collaterals are attached using the previously placed heavy suture into the allograft epicondyles. Roughening up the allograft epicondyles and suturing the host epicondylar bony wafers may assist in incorporating the ligaments to the APC. Collaterals are tightened maximally in 90 degrees of knee flexion. Supplemental cerclage wiring of the remaining epicondyle host bone can be done to reinforce the sutures.

At this point, we place morselized autograft at the host-allograft junction and attempt to suture a periosteal or synovial flap around the autograft to secure it. Additional fixation may be required if the step or oblique cuts do not impart adequate stability. We suggest using additional screws rather than a cortical strut, as the strut increases bulk to the construct and may compromise the soft tissues. Similarly, our preference is to avoid plate fixation to the allograft as multiple drill holes weaken the graft and make it susceptible to fracture or accelerated vascularization and resorption. This can be a catastrophic complication.

Overall stability of the knee is rechecked with the implants in situ. It should be anticipated early if a highly constrained implant is required based on physical exam and imaging. It is subtler in determining whether a posterior stabilized polyethylene insert or varus-valgus high-post constrained liner is required. We prefer to use the least constraint possible to avoid transfer of stress to the APC interface.

In general terms, we avoid the highly constrained implants such as a rotating hinge implant, as the force transfer to the APC junction is significant and may lead to early failure.

## 11. Extensor Mechanism Allograft

During primary or revision arthroplasty the extensor mechanism can be deficient secondary to tubercle avulsion, tendon rupture, proximal tibial bone loss, or erosion of the extensor mechanism from infection. During revision, arthroplasty scarring of the quadriceps and patellar tendon makes the extensor mechanism particularly vulnerable to disruption.

The extensor mechanism allograft is obtained from the bone bank with the complete quadriceps tendon, patellar tendon, and tibial tubercle attached. It is critical to have enough bone at the patellar tendon attachment for distal fit into the host bone.

Once the primary or revision implants are placed, the remnant of the host patella is shelled out of its periosteal sleeve. Distal tubercle is debrided, and a reverse “V” shape osteotomy is made in the area of the native tubercle. This type of osteotomy allows good press fit of the allograft and also resists proximal migration of the allograft tubercle [13].

The allograft is then placed with the host patellar remnant and allograft patella at the same level. This ideally should lie in the femoral trochlear groove of the implant. Once this height is judged, the allograft is marked at the tibial tubercle that should be very close to the native tubercle of the patient. Four small drill holes are made into the host tibia for wire passage. The graft is then shaped with a microsagittal saw to fit into the reverse “V” osteotomy site. It is press fit into the recipient site and held with transosseous cerclage wires. Proximally, the allograft quadriceps is then sutured. The allograft quadriceps tendon is attached to the remaining host quadriceps tendon in a running locked fashion with heavy, nonabsorbable suture such as fiber-wire. This is then reinforced with multiple interrupted sutures. At this point, the knee is taken through range of motion to check stability and tracking. Adjustments may still be made at this stage. If tracking and stability are adequate, multiple sutures are placed into the parapatellar tendon region. The knee arthrotomy approach is closed in the usually fashion [14].

## 12. Soft-Tissue Envelope

Closure of the wound may be challenging, and the most common reason for this is oversized allograft, followed by oversized components. To avoid this problem, careful implant and allograft selection is critical. Tibial tubercle osteotomy is attached with large fragment partially threaded cancellous screws or with transosseous wiring. Quadriceps tendon turn-down or snips are repaired with heavy suture. Closure of the parapatellar arthrotomy is done with heavy suture done in a continuous manner with reinforced interrupted sutures. Deep drains are placed at the preference of the surgeon and subcutaneous and skin layers are closed in the usual fashion. Anticipated wound closure problems should be discussed prior to surgery with your plastics colleagues. If soft-tissue coverage is a problem, rotational flaps and skin-grafting may be necessary [10].

## 13. Postoperative Care and Rehabilitation

Range of motion is a critical component of recovery, and these should be started as soon as possible provided the wound coverage is adequate and there are no extensor mechanism issues. If a tibial tubercle osteotomy or quadriceps turndown is performed, we restrict active extension for 6–8 weeks. Restrictions on weightbearing are maintained for 8 weeks followed by progressive increases to full weightbearing once graft incorporation is seen on sequential radiographs. This may take 3–6 months depending on the reconstruction and biology of the patient.

## 14. Complications

As with all complex reconstructions, preoperative planning is critical in ensuring no untoward intraoperative surprises. We strongly believe that deviating from a carefully thought-out preoperative plan may result in poor outcomes. Critical steps involve allograft and implant sizing and dealing with anticipated wound complications early and aggressively. Furthermore, optimizing the patient's perioperative health status is crucial, and this must include smoking abstinence.

Despite careful planning, complications still occur. Graft fracture, rapid revascularization, and early resorption lead to weakening of the APC and eventual failure. Another problematic scenario is a periprosthetic fracture that results in further bone loss [15]. Infections are also more prevalent in complex revision surgery. These must be aggressively treated with early debridement, antibiotics, and possible staged revision. As mentioned earlier, wound problems should be treated aggressively with appropriate consultation made to plastic surgery.

Occasionally, the combination of infection and wound problems results in an amputation although this is fortunately a rare occurrence.

## 15. Results

The use of segmental and structural allografts has been used in both contained and uncontained defects around the knee in arthroplasty for over two decades. The primary data for this comes in the setting of revision knee arthroplasty and has encouraging results. In one of the earliest papers, Stockley et al. reported 20 knees that had undergone a combination of structural allograft and morselized allograft with 85% survivorship at 4.2 years [16]. There were 2 graft fractures and 3 infections in their series. The lowest reported survivorship is that from Ghazavi et al. with only 67% survivorship at 5 years in their 30 patients [17]. However, when looking at the majority of the literature, most authors report 80–93% survivorship of their constructs at 5 years. The survivorship numbers drop off at 10 years with Clatworthy et al. showing a drop of 92% at 5 year to 79% at 10 years [18]. Reference [19] had 46 patients at 10 years with 91% survivorship for femoral head allograft in tibial defects. 

A recent publication by Richards et al. compared cohorts with severe bone loss of bone around total knee arthroplasty using femoral allograft compared with metal augments [20]. Despite the presence of more significant bone loss in the allograft group, these had better clinical outcome scores than the control cohort. This strengthens the argument for allograft use in patients with severe bone loss.

Lastly, Backstein et al. have one of the largest cohorts to date with 61 patients. The survival rate at 5.4 years was 85.2% [2]. Of note in this series, the infection rate was 6.5% (4/61); however, a high union rate of 98.4% (60/61) was seen radiographically.

## 16. Summary

Dealing with bone loss is a significant challenge to arthroplasty surgeons. We believe that structural allograft is a viable method for dealing with this problem with the added benefit of restoring bone stock. These complex procedures should be performed by surgeons with expertise in revision arthroplasty and with access to a dedicated bone bank. Allograft reconstruction is not indicated in the low demand or elderly patients who would benefit from implantation of an endoprosthesis, which allows rapid mobilization and recovery. 

The optimal allograft candidate is a young, higher demand and relatively healthy patient that is likely to require further revisions in the future and can adhere to the rehabilitation protocol. The restoration of bone stock is a key component in choosing allograft in the reconstruction. Overall, this method of treatment has good outcomes in the literature despite the complex nature of the procedures.

## Figures and Tables

**Table:** (a) AORI femoral bone loss classification

AORI femur grade	Deficit	MCL/LCL	Bone reconstruction
F1	Intact metaphyseal bone	Intact	Cement or particulate graft
F2a	Metaphyseal loss single condyle	Intact	Cement or metal augment
F2b	Metaphyseal loss both condyles	Intact	Cement, metal augment or structural graft
F3	Deficient metaphysis	Compromised	Structural allograft or segmental replacement

**Table:** (b) AORI Tibial Bone Loss Classification

AORI tibial grade	Deficit	MCL/LCL	Bone reconstruc- tion
T1	Intact metaphyseal bone	Intact	Cement or particulate graft
T2a	Metaphyseal loss med or lat Plateau	Intact	Cement or metal augment
T2b	Metaphyseal loss and lat plateau	Intact	Cement, metal augment or structural graft
T3^*⋆*^	Deficient metaphysis	Compromised	Structural allograft or segmental replace- ment

^⋆^Possible extensor mechanism compromise.

**Table 2:** Classification of Tibial and Femoral Bone Loss [8].

Type	Type of Bone Loss	Description
(1)	No notable loss of bone stock	There may be erosion of the endosteal bone, but no involvement of the cortex. There has been no migration of the primary component, and bone is largely intact.
(2)	Contained loss of bone stock with cortical thinning	The canal is widened, but there is still an intact cortical sleeve.
(3)	Uncontained (segmental) loss of bone stock involving <50% of medial and/or lateral condyle	Uncontained bone loss represents less than 50% of medial and/or lateral femoral and/or tibial condyle and is less than 15 mm in depth.
(4)	Uncontained (segmental) loss of bone stock >50% of medial and/or lateral condyle	Uncontained bone loss represents more than 50% of medial and/or lateral femoral and/or tibial condyle and is more than 15 mm in depth.
